# From nanoscale to clinic: The growing impact of nanotechnology in translational drug delivery

**DOI:** 10.5599/admet.3495

**Published:** 2026-07-27

**Authors:** Ronny Vargas, Fabiola Martos-Kikut, Noelia Martinez-Martinez, Esteban Lara-Guerrero, Catalina Lizano-Barrantes, Jorge Andrés Pacheco-Molina, Miquel Romero-Obon, Khadija Rouaz-El-Hajoui, Encarna García-Montoya, Pilar Pérez-Lozano, Carlos Suñé, Marc Suñé-Pou

**Affiliations:** 1 Department of Pharmacy and Pharmaceutical Technology, and Physical Chemistry, Faculty of Pharmacy, University of Barcelona, Barcelona, Spain; 2 Department of Pharmaceutical Technology, Faculty of Pharmacy, University of Costa Rica, San José, Costa Rica; 3 Pharmaceutical Research Institute (INIFAR), Faculty of Pharmacy, University of Costa Rica, San José, Costa Rica; 4 Department of Molecular Biology, Institute of Parasitology and Biomedicine “López-Neyra” (IPBLN-CSIC), Granada, Spain; 5 Department of Pharmaceutical Care and Clinical Pharmacy, Faculty of Pharmacy, Universidad de Costa Rica, San José, Costa Rica; 6 Pharmacotherapy, Pharmacogenetics and Pharmaceutical Technology Research Group Bellvitge Biomedical Research Institute (IDIBELL), Barcelona, Spain

**Keywords:** Nanomedicine, clinical translation, regulatory approval, innovative drug carriers, advanced therapeutics

## Abstract

**Background and purpose:**

From the early use of liposomes in the 1960s to modern nucleic acid delivery platforms, nanotechnology has shown its value in drug delivery and diagnostic applications. A wide range of nanostructures has been investigated for drug delivery, including inorganic, polymeric and lipid-based nanomaterials. While some of these nanomaterials have reached more advanced stages of clinical development, some even reaching commercialization, new alternatives are continuously being explored.

**Approach:**

This review describes the different categories of nanomaterials being used as nanocarriers and provides concrete representative examples of commercially approved products.

**Key results:**

Current trends of nanotechnology developments in the pharmaceutical market largely focus on the treatment of cancer, infectious diseases, central nervous system disorders and cardiovascular diseases. At the same time, lipid nanoparticle platforms are gaining relevance in current development pipelines. However, some of the main challenges limiting clinical translation include manufacturability, scale-up, long-term stability, regulatory uncertainty, intellectual property issues and the lack of representative preclinical study models.

**Conclusion:**

Nanotechnology has become a fundamental breakthrough in medicine, especially for advanced therapies such as gene silencing or gene editing alternatives. All things considered, no universal nanocarrier fits all biomedical applications; the evolution of the field depends on several platforms demonstrating their individual potential.

## Introduction

Nanotechnology is a multidisciplinary field encompassing the design, manipulation and characterization of structures, devices and microenvironments at the nanoscale [1,2]. Materials are generally classified as nanomaterials if at least one of their dimensions is below 100 nm [1,3], though some authors extend this definition to structures up to 300 nm, depending on the specific field of application [4,5]. Nanotechnology has diverse applications across multiple sectors, including agriculture [6], fossil fuel extraction [7], renewable energy [8,9], textiles [10], waste management [11,12], food production [13], construction materials [14] and biomedicine [15].

**Figure 1.**
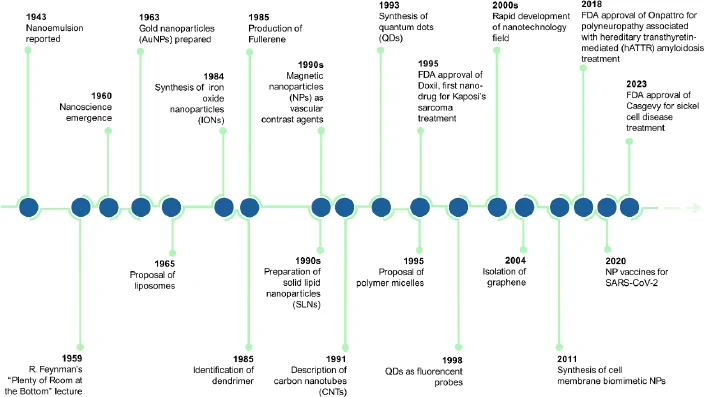
Key milestones in the history of modern nanotechnology. Following the conceptualization of nanotechnology in the 1960s, the synthesis of various nanomaterials over the next decades marked significant advancements in the field. These developments enabled diverse biomedical applications, including contrast agents, nanoconstruct-based chemotherapy and, more recently, nanoparticle-based vaccine delivery against SARS-CoV-2. Adapted from [17] under the Creative Commons Attribution 4.0 International License (CC BY 4.0), with additional modifications based on the literature reviewed in this work

Although archaeological evidence indicates that humans have utilized and harnessed the properties of nanomaterials for at least 4,500 years [2,3], modern nanotechnology is generally traced back to 1959 when Richard Feynman, later awarded the 1965 Nobel Prize in Physics, argued in his seminar lecture, "There's Plenty of Room at the Bottom” [16] that the fundamental laws of nature do not restrict our ability to manipulate matter at the atomic and molecular scale; rather, the limitation lies in the absence of suitable tools and techniques [3]. Indeed, over the past six decades, advancements in instrumentation have propelled nanotechnology into a rapidly expanding scientific discipline [17]. Figure 1 highlights some of the most significant milestones in the evolution of modern nanotechnology.

Consistently, nanotechnology has emerged as a promising approach for developing advanced drug delivery systems. From the early use of liposomes in the 1960s to modern nucleic acid delivery platforms, nanotechnology has proven its value not only in drug delivery and diagnostics but also in clinical applications. A major milestone in nanomedicine was the U.S. Food and Drug Administration’s (FDA) 1998 approval of Doxil®, a liposomal formulation of doxorubicin initially indicated for Kaposi’s sarcoma [2]. Encapsulation enhanced its pharmacokinetic profile, improving safety and efficacy over free doxorubicin [18,19]. More recently, the clinical success of therapeutics based on small interfering RNA (siRNA) [20,21], gene-editing treatments [22,23], and messenger RNA (mRNA) vaccines for COVID-19 [24] underscores the transformative role of nanotechnology in modern medicine. Besides drug delivery, nanotechnology has demonstrated considerable potential in biosensors, tissue engineering, nanobiotechnology, and diagnostic tool development [25].

Through precise engineering, nanotechnology facilitates the combination of therapies, the circumvention of biological barriers and the controlled modification of drug properties [26]. As a result, nanoengineered systems improve drug stability and extend circulation time in the body [27]. Additionally, nanomedicine formulations allow precise control over dosage, accumulation and, in some cases, drug release site [25,27]. Integrating biofunctional structures into nanomaterials enables active targeting toward specific cells or tissues [28,29]. The biomedical impact of nanotechnology stems from its functional characteristics, which align with biological processes occurring at the nanoscale [30].

Nanoparticles have demonstrated their ability to transport a wide range of molecules, from small molecules to proteins and nucleic acids [31,32], as well as hybrid drug-diagnostic systems [33,34]. This versatility has enabled their investigation for multiple preclinical applications, reinforcing nanotechnology’s promise in biomedical development. Nanomaterial-based delivery systems have been most extensively applied in oncology [26], primarily due to their ability to minimize side effects and enhance accumulation in tumour tissues [35,36]. Their potential has also been widely investigated in other therapeutic areas, including infectious diseases [37-39], cardiovascular disorders [40-43], metabolic disorders [44-47], neurological conditions [48-52] and rare diseases [53-56], among others.

Beyond the extensive preclinical research in nanomedicine development, many studies have progressed to clinical evaluation or are approaching this stage [48-50,57-61]. A search of the ClinicalTrials.gov database in December 2025 identified 815 registered studies involving nanoparticles, of which 135 had reported results and 252 were active, predominantly in Phase I and Phase II trials [62]. Complementing these clinical efforts, around one hundred nanomedicine formulations have achieved regulatory approval, providing concrete evidence of the translational potential and practical impact of nanotechnology in medicine [63,64].

## Types of nanomaterials used as drug delivery systems

**Figure 2.**
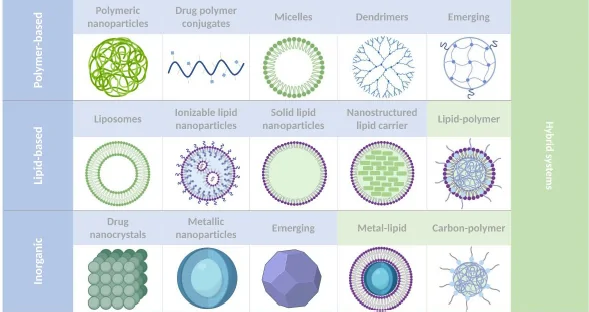
Types of nanomaterials used as drug delivery systems. Grouped into four main categories: polymeric nanoparticles, lipid-based nanoparticles, inorganic (also referred to as non-polymeric) nanoparticles and hybrid systems. Created by the authors with BioRender.com based on the synthesis of the literature reviewed in this article

A wide range of nanostructures has been investigated for drug delivery applications. While some of these nanomaterials have reached a more advanced stage of clinical development, new alternatives are continuously being explored, with the potential to enrich and diversify therapeutic options in the future. These nanomaterials differ in composition, structure and functionality, features that confer specific advantages for various biomedical applications [15,26]. Figure 2 illustrates some of the traditionally used nanomaterials for drug delivery, grouped into four main categories. In the following sections, each class of material is addressed individually, with particular emphasis on subtypes that have demonstrated more advanced clinical translation. In addition, emerging materials are briefly discussed to highlight their potential and to provide a forward-looking perspective on future developments.

### Polymeric-based nanomaterials

Polymers are distinguished from other drug-delivery vectors, such as lipids or metals, primarily because of their versatility [65,66]. The ability to incorporate diverse functional groups, together with precise control over size and shape, confers polymers a broad range of functionalities and applications, as is described in the subsequent section [66,67].

### Polymeric nanoparticles

Polymeric nanoparticles, composed of either synthetic or natural polymers, assemble biocompatible and biodegradable materials that allow precise control over both the release of encapsulated therapeutic agents and surface drug delivery. Polymeric nanoparticles can be prepared using various methods from either natural polymers, such as chitosan, gelatine, albumin, or alginate, or synthetic polymers such as poly (D,L-lactic acid), poly(D,L-lactic-co-glycolic acid) (PLGA) and poly(ε-caprolactone) and their copolymers [68,69]. These materials can modulate drug release in response to environmental stimuli, such as temperature or pH [70]. The degradation of certain polymers (*e.g*. PLGA) yields non-toxic products like CO₂ and H₂O, which are naturally eliminated, thus contributing to their favourable safety profiles [71].

From a translational perspective, the most solid example of the clinical advancement of polymeric nanoparticles is Abraxane®, an albumin-bound nanoparticle formulation of paclitaxel, approved alone or in combination, for the treatment of metastatic breast cancer, non-small cell lung cancer, and metastatic pancreatic adenocarcinoma, which has demonstrated enhanced tolerability and clinical utility compared to free drug administration [72,73]. Although proteins are natural polymers, protein-based nanoparticles such as Abraxane® are often classified in the literature as a distinct subtype of nanoparticles (protein nanoparticles).

Polymeric nanoparticle systems have attracted considerable attention in preclinical and clinical research and have been explored as carriers for small-molecule drugs, peptides, and nucleic acids, as well as for theragnostic applications. Polymeric nanoparticles hold significant promise for transforming cancer therapy into more effecttive and personalized treatments through ongoing clinical evaluation and material innovation [65,66,74,75]. For an in-depth description of polymeric and polymer-related nanoparticles, readers may refer to previously published reviews [65,67,76,77].

### Drug-polymer conjugates

A different type of polymer-based platform involves the covalent linking of polymers to small molecules. This chemical conjugation increases molecular weight, thereby altering distribution and cellular affinity. Additionally, these conjugates improve stability, solubility and permeability. In the presence of pH-sensitive linkages, drug-polymer conjugates can release the molecules at specific sites, enhancing targeted delivery [68,78].

A clinically relevant example of this strategy is FYARRO®, an albumin-bound conjugate of sirolimus approved for the treatment of malignant perivascular epithelioid cell tumours. This conjugation enables the systemic delivery of a poorly soluble molecule while improving tolerability and clinical utility. Other examples include early clinical candidates such as PK1 (HPMA-doxorubicin) and NC-6300 (epirubicin-polymer conjugate), evaluated in Phase I clinical studies for solid tumours. Beyond oncology, conjugates such as APL-2 (pegcetacoplan) have advanced to Phase III trials in haematological disorders, further illustrating the translational breadth of polymer-drug conjugation strategies [79].

### Micelles

Micelles are colloidal structures formed through the self-assembly of amphiphilic molecules in solution, utilized for their ability to enhance the solubility and stability of hydrophobic drugs. The micellar constituents are organized into spherical structures, with hydrophilic groups surrounding the hydrophobic core, thereby encapsulating hydrophobic drugs within their core [68,80].

Genexol® is a polymeric micellar formulation of paclitaxel approved for the treatment of breast cancer, which has demonstrated enhanced antitumour activity and increased tumour tissue accumulation compared with free paclitaxel and earlier paclitaxel formulations. In addition, this micellar system has shown encouraging clinical outcomes in multiple trials for the treatment of breast, lung, and ovarian cancers [65,81,82]. Other advanced micellar formulations under clinical evaluation include NK105, NK012, NC-6004, NC-401, NK911, CPC634, and Nanoxel M®, which are currently in different phases of clinical development and have reported promising improvements in safety and efficacy profiles across various oncological indications [83,84].

### Dendrimers

Dendrimers are highly branched and compartmentalized nanopolymers that exhibit a uniform and well-defined three-dimensional structure [64,85]. They typically range in size from 1 to 5 nm and are capable of efficiently encapsulating molecules within their internal cavities. The terminal groups on dendrimer surfaces can be chemically modified to enhance biocompatibility, solubility and permeability, or to enable active targeting [68]. In preclinical studies, dendrimers have been explored as carriers for both small-molecule drugs and biomacromolecules, including nucleic acids, peptides, and vaccines. Structurally, they are commonly synthesized from synthetic monomers or copolymers such as poly(amidoamine), poly(propyleneimine), and poly(ethylenimine), although dendritic or hyperbranched architectures based on biopolymers have also been reported [86,87].

Advancing from preclinical research, dendrimers have demonstrated clinical translation, with several systems progressing to marketed products and advanced clinical trials. Dendrimer-based formulations are currently marketed for the prevention and treatment of viral and bacterial infections, including sexually transmitted diseases and respiratory viruses, such as VivaGel™ for bacterial vaginosis and human immunodeficiency virus prevention, and VIRALEZE™ as an intranasal antiviral formulation against respiratory viruses [88,89]. In parallel, multiple dendrimer-based therapeutics have advanced into Phase I-II clinical trials for oncological and inflammatory indications [90]. More recently, dendrimer platforms have expanded into the field of neuroinflammation and neurodegenerative disorders, with active clinical studies evaluating their potential both as therapeutic agents and, to a lesser extent, as diagnostic tools capable of reaching the central nervous system [91].

### Emerging polymeric-based nanomaterials

Other polymeric-based nanomaterials have demonstrated strong preclinical promise; however, their clinical translation remains limited to date. Nevertheless, these platforms represent opportunities for further development due to specific advantages over conventional nanostructures. Representative examples include nanogels, polymersomes, and stimuli-responsive or polymer-hybrid nanocarriers, including polymer-lipid hybrid systems [68,92,93]. As the present work focuses on nanocarriers with more advanced clinical translation, these systems are not discussed in detail here. Readers interested in these platforms may refer to the extensive body of existing literature on the subject [94-96]. Further research aimed at identifying and overcoming current limitations, as well as expanding the understanding of their applicability, may ultimately support their progression toward clinical adoption of these emerging nanomaterials [94,97].

### Inorganic nanomaterials

Inorganic nanomaterials have attracted considerable interest in the biomedical field due to their unique physicochemical, electromagnetic, optical and mechanical properties [17,98]. Although the application of inorganic nanomaterials as delivery agents for antibodies, nucleic acids, peptides and small molecules has been explored, their clinical translation remains limited, primarily due to concerns regarding long-term safety [98,99].

### Drug nanocrystals

Nanocrystals are primarily composed of crystalline active pharmaceutical ingredients, with minimal use of stabilizing agents at low concentrations. These systems are applied to poorly soluble drugs, where their nanocrystalline form improves apparent aqueous solubility and bioavailability due to the improved surface-to-volume ratio. These features allow nanocrystals to achieve higher drug loading and concentration efficiency [68,100]. Some examples of marketed products consisting of nanocrystal formulations include Cabenuva® (Cabotegravir), Focalin XR® (Dexmethylphenidate hydrochloride) or IVEMEND® (Fosaprepitant) [101].

Nanocrystal formulations have shown success enabling clinically meaningful shifts in therapeutic use by unlocking new dosing paradigms or routes of administration. For example, antiretroviral nanocrystal/nanosuspension formulations (*e.g.* cabotegravir and rilpivirine) were specifically designed to support long-acting injectable regimens, addressing adherence limitations associated with daily oral therapy [102,103]. Similarly, nanoparticulate fenofibrate formulations reduce food-related variability in exposure, allowing more flexible dosing [104]. Beyond these benefits, nanocrystal reformulation represents a lifecycle-management strategy that may facilitate drug repurposing by overcoming solubility- and exposure-limited development [105,106].

### Metallic nanoparticles

These nanomaterials have diameters between 1 and 100 nm and are typically composed of metals such as gold, cobalt, nickel, iron and their oxides. They can be readily synthesized and modified to decorate their surface with various molecules, including small molecules and biomolecules such as DNA and proteins [68,107]. These particles are biocompatible and stable. In addition, their magnetic properties allow them to be guided to specific locations in the body using external magnetic fields [98]. Metallic nanoparticles have both therapeutic and diagnostic applications, primarily in the field of oncology [98,108].

Approved applications of this type of nanocarrier include Nanobiotix®, which comprises hafnium oxide nanoparticles designed to enhance radiotherapy by promoting higher local energy accumulation in tumour tissues [101]. Another example is Nanotherm®, a formulation based on superparamagnetic iron oxide nanoparticles that enables magnetic hyperthermia; once the nanoparticles accumulate within the tumour environment, applying a magnetic field induces localized heating, leading to thermal destruction of tumour tissue [109].

### Emerging inorganic nanomaterials

Despite extensive preclinical development and attractive physicochemical properties, several inorganic nanomaterials have been widely studied but have not yet achieved widespread clinical translation as drug delivery platforms. Among these, silica-based nanoparticles, carbon nanotubes, nanodiamonds, and quantum dots have received considerable research attention due to their versatility and tunability, which derive from their distinct structural, physical, and functional characteristics; however, their clinical use remains limited. Collectively, promising candidates illustrate the gap between technological promise and clinical implementation within inorganic nanomaterials [108,110-114].

### Lipid-based nanomaterials

As discussed in the *Evolution of the pharmaceutical market for nanoparticles* section, current market trends indicate a growing relevance of lipid nanoparticles in clinical applications. This growing interest reflects their excellent biocompatibility and inherent compatibility with biological tissues, stemming from their similarity to cell membranes [115-117].

### Liposomes

Liposomes are spherical structures with a lipid bilayer surrounding an aqueous core. They were the first lipid-based nanocarriers described and are perhaps the most representative of them [118,119]. Their structure allows them to encapsulate both hydrophilic and hydrophobic molecules, incorporated within the aqueous core or the lipid bilayer, respectively. Liposomes can fuse with cell membranes, releasing their contents directly into the cytoplasm, making them effective carriers for selective therapy delivery. Liposomes may be unilamellar or multilamellar, depending on the number of lipid bilayers they contain [68,120].

These carriers have been employed in therapeutic applications such as tumour detection and treatment, antibacterial therapy, vaccination and even targeted drug delivery to the brain. Surface modifications, such as coating with functionalized polymers or polyethylene glycol (PEG) chains, facilitate targeted distribution and improve circulation time in biological systems [68].

As previously mentioned, Doxil® represents a paradigm shift in the clinical impact of liposomal formulations, and, more broadly, of nanotechnology, on drug delivery. By significantly improving the pharmacokinetic profile and reducing toxicity relative to free doxorubicin, the liposomal nanocarrier directly enhanced clinical tolerability and enabled the expansion of therapeutic indications for a well-established anticancer agent [18,19,121]. Other examples of well-established liposomal clinical platforms include Ambisome® (amphotericin B), which is considered the standard medication for severe systemic fungal infections [122], or Vyxeos® (daunorubicin and cytarabine) approved for the treatment of acute myeloid leukaemia [123], among others.

### Ionizable lipid nanoparticle

Ionizable lipid nanoparticles (i-LNPs) represent a more recent class of delivery systems that have gained increasing relevance alongside the development of nucleic acid delivery technologies [124,125]. Their use has become so standardized for this purpose that, in this context, they are often referred to simply as lipid nanoparticles (LNPs) [126-131]. They constitute the most clinically advanced delivery systems for maintaining the stability of therapeutic nucleic acids and enabling the efficient delivery of siRNA and mRNA [132-135], as well as in CRISPR-Cas9 and other gene-editing applications [136-138].

Because of their relative novelty, some reviews in the field of lipid nanoparticles do not yet recognize them as a distinct category, which can lead to LNPs being mistakenly classified as liposomes or even NLCs [68,139]. However, i-LNPs display clear structural and functional distinctions from other lipid-based nanoparticles [125,140]. They are composed of four main lipid components: ionizable lipids, phospholipids, cholesterol, and PEGylated lipids [129,141].

At acidic pH, ionizable lipids carry a positive charge, enabling electrostatic complexation with negatively charged nucleic acids by pH adjustment during manufacturing. However, they are neutral at physiological pH, which helps minimize toxic effects during systemic circulation [124,125,129]. After internalization and subsequent endosomal acidification, i-LNPs structure becomes unstable due to electrostatic repulsion and therefore releases their cargo into the cytoplasm via endosomal escape, a crucial step that protects RNA from degradation and allows the nucleic acid to reach its intracellular target [128,142,143].

Illustrating the advancement of this specific type of nanovehicle, Onpattro®, the first siRNA-based product delivered *via* ionizable lipid nanoparticles, was approved in 2017 for the treatment of hereditary transthyretin amyloidosis [144,145]. In 2020, Comirnaty®, an mRNA vaccine against SARS-CoV-2, received emergency approval for global immunization in response to the COVID-19 pandemic [146,147]. In 2023, Casgevy® received regulatory approval as the first CRISPR-Cas9-based gene-editing therapy for sickle cell disease, reinforcing the position of ionizable lipid nanoparticles as clinically validated platforms for advanced medicines [22].

### Lipid nanocarriers with limited clinical translation

SLN and NLC were developed earlier than ionizable lipid nanoparticles and have been extensively investigated across diverse preclinical applications and drug classes [119,148,149]. However, their clinical translation for systemic drug delivery has remained limited, despite the large number of preclinical studies and patented formulations [150-152]. This has been mainly attributed to crystallinity-driven drug expulsion and physical instability, including polymorphic transitions and formulation-dependent leakage, which are partially mitigated in NLC [153,154]. In addition, challenges related to long-term stability, scale-up, and reproducibility remain significant barriers compared with more industrially mature platforms such as liposomes and ionizable lipid nanoparticles [155-157].

### Hybrid systems as emerging nanomaterials applied to nanomedicine

Hybrid nanoparticles, which merge materials of diverse origins to optimize their characteristics, are also found in various applications [158]. For example, hybrid nanoparticles may be metal-lipid [159], lipid-polymer [160] or carbon-polymer [161]. These offer the advantage of creating synergy among the components, improving attributes such as stability, adaptability of manufacturing methods, drug release efficiency and biocompatibility, thereby optimizing performance in therapeutic or diagnostic applications [158,162]. Over the coming years, research and applications involving these materials are expected to expand, potentially leading to the emergence of new nanomaterials for biomedical exploration [163,164].

In general, each type of nanomaterial is characterized by unique properties that confer suitability for specific diagnostic or therapeutic applications [25,165]. The engineering and strategic exploitation of these properties have enabled the overcoming of translational barriers, driving the progression from experimental concepts to clinically validated therapeutic options with impact on disease treatment and the pharmaceutical market.

## Evolution of the pharmaceutical market for nanoparticles

**Figure 3.**
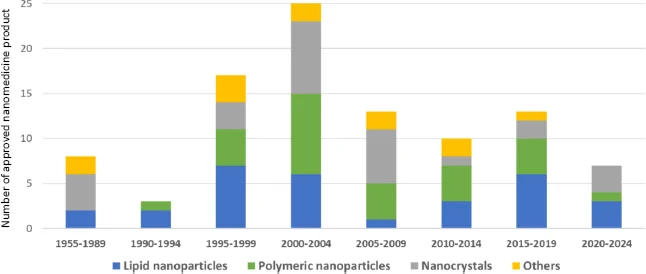
Evolution of nanotechnology-based drug approvals (1955-2024). Number of approved nanomedicine products by material class across successive time periods. The data show an increase in approvals from the mid-1990s to the early 2000s, followed by relatively stable approval rates in later years, reflecting the establishment and consolidation of clinically successful nanomedicine platforms. Figure prepared by the authors based on information from references [101,121,169,170]

The earliest approvals of drugs applying nanotechnology date back to the 1980s, 1970s, and even the 1950s, with the authorization of several medicines whose characteristics and efficacy were based on their composition using nanocrystals [68,166-168]. Figure 3 illustrates the evolution of nanotechnology-based drug approvals over the years, while references [101,121,169,170] provide detailed lists of the approved products.

A report by Clogston and colleagues [147] provides an industry perspective on the current state of nanomedicine drug products based on the PharmaCircle™ database of pharmaceutical product pipelines and complemented by insights from companies active in nanotechnology-based pharmaceutical innovation. According to this industry-based analysis, nanocrystals represented the most prevalent nanomaterial type (38 %), followed by lipid nanoparticles (32 %) [147]. These observations are broadly consistent with U.S. Food and Drug Administration (FDA) data compiled between 1973 and 2015, in which liposomes (33 %) and nanocrystals (23 %) have been reported as the two most prevalent nanomaterial categories [171]. Minor differences in percentages may reflect sample representativeness, evolving formulation trends, or variations in classification criteria over time, particularly as lipid nanoparticle classifications have expanded beyond earlier liposome-focused categories.

Importantly, Clogston and colleagues [147] reported that commercially established products represent only 11 % of the overall nanomedicine portfolio, while most developments remain in preclinical or early clinical stages. Specifically, preclinical candidates account for 49 % of the lifecycle pipeline, whereas Phase I, II, and III clinical studies represent 12, 5 and 5 %, respectively [147].

Expanding the analysis beyond commercially established products to the broader development landscape reveals a marked shift in nanomaterial composition, with lipid-based nanoparticles increasing from approximately 28 to 32 % among marketed nanomedicines to 58 % of the overall pipeline [147]. This trend may reflect an increasing focus on lipid-based systems in current nanomedicine development.

From a therapeutic standpoint, cancer remains the leading focus of nanomedicine development. Nevertheless, an emerging trend is the diversification towards targeting infectious diseases and inflammatory, immune, and pain disorders. Emerging interest concentrates on central nervous system conditions, suggesting a more recent and rapidly growing area of therapeutic interest [147,171,172].

The market trends over the past three decades, together with advances in innovative therapies and increasing regulatory acceptance, suggest a promising future for nanotechnology-based developments [25,141,147]. Despite the high costs associated with these products [173,174], pharmaceutical companies are investing heavily in the development of nanotechnology platforms, indicating that nanotechnology will continue to have a major impact on the pharmaceutical market [121].

**Figure 4.**
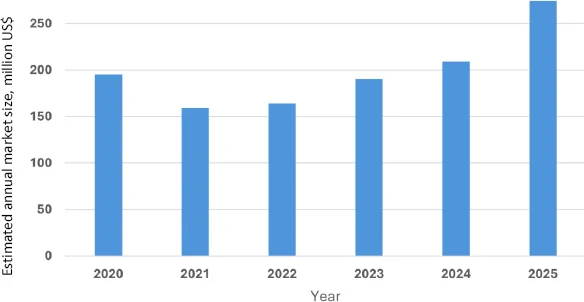
Estimated evolution of the global nanomedicine market (2020-2025). Approximate annual market size estimates (USD millions) compiled by the authors from published studies and industry reports [175-180]. The market value observed in 2020 may reflect the immediate impact of the COVID-19 pandemic, likely associated with nanotechnology-based vaccine platforms

The increasing regulatory acceptance and expansion of nanomedicine pipelines are reflected not only in current market dynamics, but also in projections for the coming years. Figure 4 summarizes the approximate annual value of the nanotechnology-based therapeutic market over recent years, compiled from industry reports and published sources. For the upcoming years, the global market value estimates for these products are projected to reach up to USD 350 billion per year between 2026-2030 [63,170], while more aggressive scenarios indicate a potential market size above USD 420 billion annually by the end of this decade [175]. Collectively, the available evidence underscores the considerable potential for nanotechnology to continue impacting the pharmaceutical landscape, as translational barriers are progressively identified and addressed.

## Current challenges for the clinical advancement of nanoparticles

Despite advances in the nanomedicine market, there is still a considerable imbalance between academic research and clinical application in the field of nanomedicine [121]. Because nanomaterial performance is tightly linked to physicochemical properties, clinical translation depends on the development of robust, reproducible manufacturing and characterization processes [30]. At the same time, biological interactions, such as immune responses, and environmental considerations pose substantial challenges to nanomedicine development [25,176,181]. In addition, limited harmonization among regulatory frameworks across agencies may increase the complexity of clinical translation [57].

According to leading companies in the biotechnology sector, as reported by Clogston *et al.* [147], the main barriers limiting advancement include challenges related to manufacturability and scalability (40 %), development timelines (24 %) and installed capacity (18 %). Building on this industrial perspective, the following sections complement this view by including challenges discussed in the academic literature, offering a more comprehensive overview.

### Challenges in industrial-scale manufacturing

A major challenge in the clinical translation of nanoparticle-based therapeutics concerns large-scale production, which is often hindered by difficulties in process standardization. This affects the reproducibility and economic feasibility of industrial nanoparticle manufacturing. The inherent complexity of these drug delivery systems, typically composed of multiple components, introduces both technical and economic barriers that slow the development of marketable products [141,182-184].

Specific issues include inadequate in-process quality control, the absence of industrial-scale equipment, difficulties in purification, high production costs, low yield, limited infrastructure and technical expertise, all of which are further aggravated by insufficient financial investment [185].

While efficient techniques already exist for the large-scale manufacturing of materials such as liposomes, the challenges increase significantly as systems become more complex. The incorporation of surface modifications, targeting elements or the encapsulation of multiple therapeutic agents requires several production steps. This not only increases costs but also complicates quality control and compromises stability [182,185]. Furthermore, investment in custom instrumentation, manufacturing equipment and facilities may be prohibitively expensive for some companies or may require staggered investment strategies contingent upon achieving incremental clinical development milestones, thereby extending timelines [182].

The challenges associated with industrial-scale production are not only critical themselves but also influence other obstacles in the clinical translation of nanoparticles. In particular, the ability to scale up functionalization processes is essential for innovations that aim to modify nanoparticles for better targeting or altered biodistribution [186].

In addition, scaling up manufacturing processes in compliance with good manufacturing practices (GMP) is critical to ensure both technical robustness and regulatory conformity, thereby guaranteeing the consistent quality and safety of the final drug product [187]. This interdependence emphasizes the need for an integrated and multifactorial approach to address the complexities of nanomedicine industrialization.

### Challenges related to long-term stability

Significant challenges are associated with the long-term stability of nanostructured preparations, as they may lose activity within relatively short timeframes, ranging from weeks to months. Currently, some of these formulations require storage and transport under frozen conditions to maintain their functionality. Overcoming these limitations is essential to reduce costs and logistical complications related to cold-chain management [188,189]. Research on lyophilization has shown promising results as a potential solution, allowing these nanoparticles to be stored at ambient or even elevated temperatures while retaining bioactivity [190-192].

### Challenges related to selective accumulation

Selective accumulation of nanoparticles remains a major challenge, as a considerable fraction of nanomedicines tends to accumulate uncontrollably in non-target organs, thereby reducing therapeutic efficiency. Elucidating the mechanisms governing nanoparticle transport may guide the development of formulations with improved accumulation efficiency at specific sites and, thereby, enhance clinical translation [193].

The surface of nanoparticles can be modified with ligands that recognize and bind to specific receptors present in target tissues, thereby facilitating accumulation at the desired site [194,195]. However, the potential of functionalization for active targeting may be limited by a lack of selective expression of the receptors targeted by nanoparticles. Additionally, the added ligands may be masked by the protein corona once in systemic circulation, concealing their targeting effects [193].

Factors such as the composition, size and shape of the base nanoparticles also influence their biological behaviour, including their blood circulation profile, site-specific accumulation and ability to penetrate tissues and specific cells [116,196].

Active targeting also depends on overcoming challenges such as nanoparticle stability in circulation, evasion of immune responses [197,198] and the ability to traverse biological barriers such as the blood-brain barrier [172,193]. It also involves the development of systems capable of responding to microenvironmental changes or external stimuli to achieve controlled drug release [70]. The complexity of addressing these challenges is further amplified by interpatient and tissue variability, which can affect therapeutic uniformity and efficacy [199].

### Challenges in preclinical predictive models

One of the major challenges in the clinical translation of nanomedicine lies in the lack of preclinical models that accurately represent the heterogeneity of human diseases and their specific interactions with nanoparticle systems. This gap frequently results in a disconnection between preclinical outcomes and clinical performance, particularly affecting the early evaluation of safety and therapeutic efficacy [200,201].

It has been observed that nanoparticle-based drugs tend to exhibit lower predictability in their clinical behaviour during preclinical evaluation than conventional pharmaceuticals. This variability may arise from the critical influence of pharmacokinetic parameters, tissue distribution, accumulation and penetration at the target site and drug release within the target tissue, or even within target cells [202].

The chronic toxicity of nanomaterials, both at the cellular and systemic levels, must be carefully monitored to ensure the absence of long-term adverse effects. Such toxicity may vary depending on particle size, shape and composition, highlighting the need for strategies to evaluate and mitigate these potential risks [185,203].

Emerging evaluation strategies, such as organ-on-a-chip platforms, hold promise for reducing the gap between current preclinical models and in vivo behaviour [204], thereby contributing to improved clinical translation because of better predictive models.

### Regulatory approval challenges

Within the specific area of regulatory approval, several authors have noted that regulation surrounding nanoparticles faces major challenges due to the lack of clear standards and adequate regulatory frameworks. Globally, regulatory agencies such as the FDA and the European Medicines Agency (EMA) still operate under traditional frameworks that may not be fully sufficient to ensure the quality and safety of nanomaterials. However, excessive regulation can also hinder innovation and increase approval costs [57,185].

International cooperation is essential to establish global standards for the regulation of nanoparticles in the biomedical field, ensuring their production under GMP. In addition, new analytical techniques are needed to assess their physical properties, along with standardized procedures to guarantee efficacy and safety [185].

The FDA maintains that its current regulatory framework is sufficiently robust and flexible to accommodate materials of different nature, including nanomaterials. These are therefore evaluated under existing regulatory guidelines [183]. Nonetheless, the agency acknowledges the need to reassess current classification systems and to develop dedicated tools for this field. Among its recommendations are the establishment of close collaboration between industry, academia and international organizations, the development of specific toxicological assessment assays and a deeper understanding of the correlation between physicochemical properties and in vivo biological behaviour [183].

### Intellectual property challenges

Intellectual property challenges in nanomedicine stem from the fact that these developments often involve multiple aspects that may be subject to protection: (i) the encapsulated drug; (ii) the carrier technology; and (iii) the combined characteristics of the drug-carrier system, along with any additional modifications to the base vehicle. This complexity poses challenges in patent approval, especially when novel components are combined with previously protected elements. Such scenarios often necessitate cross-licensing agreements and multiple overlapping patents covering the same technology [185,205].

This issue can be exacerbated by the indiscriminate accumulation of patent filings, which may delay innovation approval, a practice known as patent thickets, and by the issuance of invalid patents. The complexity of intellectual property management in nanotechnology can lead to costly litigation that delays commercialization. Therefore, the development of international standards and policies specifically addressing intellectual property issues in health-related nanotechnologies is urgently needed [185,206].

**Figure 5.**
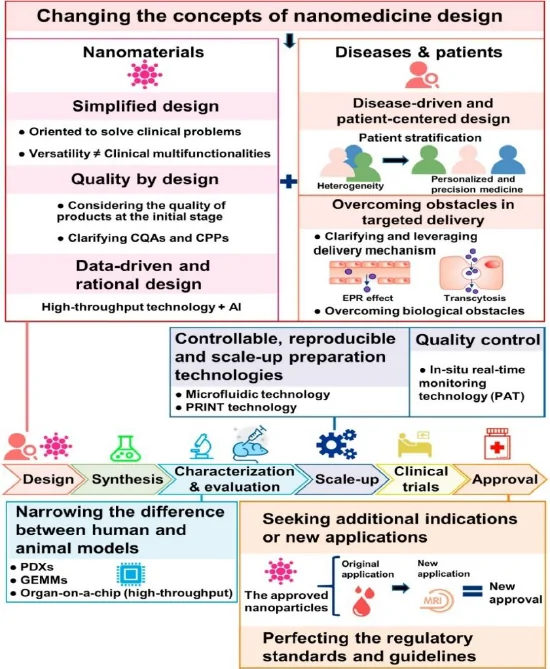
Perspectives for Addressing the Challenges in the Clinical Translation of Nanomedicine. The figure summarizes the integrated framework proposed by Shan et al. to address key translational challenges across the nanomedicine life cycle, spanning rational and patient-centred design, scalable and reproducible manufacturing, improved preclinical model relevance, regulatory alignment, and expansion toward new clinical indications. Reproduced from [193] under the Creative Commons Attribution 4.0 International License

Translational barriers in nanomedicine involve complex, multifactorial aspects that demand a multidisciplinary approach [183]. Shan et al. propose a framework to address these challenges by organizing them into strategic blocks aligned with the drug life cycle. This framework, shown in Figure 5, emphasizes the need to integrate technological innovation with appropriate regulatory frameworks. Together, these elements highlight the importance of cross-disciplinary integration to effectively advance nanomedicine from the nanoscale to the clinic.

## Discussion

In previous sections, we have reviewed evidence supporting that nanomedicine is not only an academic trend or a future promise. Instead, it is a validated and increasingly diversified drug delivery strategy. Evidence shows that it not only impacts therapeutic availability but has also impacted the technological and economic landscape of the pharmaceutical industry.

From a historical perspective, early nanotechnology-based medicines entered the market before widely cited milestones; however, certain approvals became emblematic as they consolidated a broader translational narrative. Doxil®, for example, was not merely a liposomal formulation but a clinical proof that nanocarriers could meaningfully reshape pharmacokinetics, tolerability, and therapeutic utility, helping to legitimize nanomedicine as a viable development strategy [166]. This probably explains the predominance of Doxil® when citing nanotechnology milestones. More recently, the clinical success of nucleic-acid delivery platforms further strengthened this paradigm by demonstrating that nanocarriers can enable therapeutic alternatives that are otherwise impractical due to instability, biodistribution constraints, or intracellular delivery barriers [207,208].

The diversity of platforms employed as well as their temporal evolution indicates that no single nanocarrier is universally optimal across time, therapeutic applications or technological context. Different types of nanocarriers adapt to different conditions: payload class, route of administration, target tissue accessibility, dosing frequency, and safety margins. These carriers differ in stability, formulation complexity, and susceptibility to variations in biological performance. Therefore, this evidence suggests that while translation is reachable for different types of platforms, their progression favours fit-for-purpose designs that integrate clinical demands with feasibility considerations, rather than the most sophisticated (or simplified), or fashionable vehicles. In other words, this suggests that no universal nanocarrier fits all biomedical applications, and the evolution of the field depends on several platforms demonstrating their individual potential.

Despite market growth and increasing regulatory acceptance, the costs associated with most nanotechnology-based medicines remain an important barrier to access, potentially limiting both development capacity and patient availability. This challenge is particularly evident in advanced therapies, such as gene-silencing and gene-editing treatments. However, it is important to analyse whether the high costs associated with some of these therapies reflect the intrinsic development or manufacturing expenses, or whether they are primarily driven by niche pricing related to rare diseases, small patient populations, and other pharmacoeconomic considerations, regardless of whether the therapies are advanced or conventional [209-211].

Although detailed manufacturing methods fall outside the scope of this review, manufacturability remains one of the most decisive filters for clinical advancement. The production method strongly influences the nanoparticles attributes that are critical to their biological behaviour, but also the scalability, batch-to-batch reproducibility, scalability, and directly the cost of production. Therefore, the reader is encouraged to consult the available literature on the technological constraints and advances in nanotechnology manufacturing.

The analysis of the existing challenges suggests that translation might be facilitated by the synergistic convergence of scientific, technological, regulatory, and economic actors. In this context, progress in each field must be integrated. Translation depends on rational, disease- and patient-centred designs, and also on evaluation and regulation frameworks aligned with the clinical and industrial context. The most meaningful advances will arise from cross-disciplinary integration, where advances at the nanoscale will continue growing and maturing into more and more clinically translated therapies.

Despite these constraints, the number of marketed nanomedicine products serves as both validation and inspiration for continued development. Their successful clinical translation demonstrates that during the last decades, nanomedicine has driven a paradigm shift in pharmaceutical research and development, transforming early promises into industrially mature and clinically impactful applications.

## Abbreviations

EMA: European Medicines Agency
GMP: good manufacturing practices
i-LNPs: ionizable lipid nanoparticles
LNPs: lipid nanoparticles
mRNA: messenger RNA
NLC: nanostructured lipid carriers
PLA: poly (D,L-lactic acid)
PLGA: poly(D,L-lactic-co-glycolic acid)
PCL: poly(ε-caprolactone)
PEG: polyethylene glycol
siRNA: small interfering RNA
SLN: solid lipid nanoparticles
FDA: U.S. Food and Drug Administration
